# Effects of Blending on Phenolic, Colour, Antioxidant and Aroma Components of Cabernet Sauvignon Wine from Xinjiang (China)

**DOI:** 10.3390/foods11213332

**Published:** 2022-10-24

**Authors:** Huan Wang, Yuanyuan Miao, Xiaoyu Xu, Piping Ye, Huimin Wu, Bin Wang, Xuewei Shi

**Affiliations:** College of Food Science, Shihezi University, Shihezi 832000, China

**Keywords:** blending, variety, organic acids, phenolic compounds, antioxidant activity, color, volatile components

## Abstract

High-quality wines in industrial winemaking frequently require a professional winemaker to make adjustments according to the wine of single-batch fermentation. Blending can improve the chemical composition and certain organoleptic properties of wine, promote copigmentation, and increase the complexity of the wine body and aroma. In this study, high-performance liquid chromatography (HPLC) and headspace solid-phase microextraction with gas chromatography coupled to tandem mass spectrometry (HS-SPME-GC-MS/MS) were used to study the effects of adding 20% of Merlot, Marselan, Syrah and Pinot Noir and different blending methods on the nutritional, taste, color and aroma components of Cabernet Sauvignon wine. The results showed that the highest total phenols and flavonoids, the greatest content of antioxidant characteristics, the optimal color according to the parameter of T, red% and blue% and the most abundant aroma were observed both in CGM (grape blending Cabernet Sauvignon and Merlot) and CGS (grape blending Cabernet Sauvignon and Marselan), thus indicating the higher quality and complexity of these wines. In addition, the co-grapes treatment afforded more color and hue value than co-wines, which indicates co-grapes had more stable and more varied colors than co-wines. Our findings provide theoretical support for improving wine quality and craftsmanship.

## 1. Introduction

Wine is a very complex mixture of compounds such as carbohydrates, polyphenols, organic acids, proteins, minerals and more [1]. It can be divided into white wine, rosé wine and red wine by color. Dry red wines dominate the market due to their formidable style and abundant body. Different brewing grapes have their own unique vinification characteristics and individual styles [2,3]. In addition, the style of wine also received the influence of the geographical environment [4].

What is memorable about wine is its consummate quality, such as taste, nutrition, color and aroma, etc. [5]. Organic acids are important compounds that affect the coordination and stability of wine. Excessive low acidity will lead to a dull taste and poor storage of wine, and acidity that is too high will result in the rough and emaciated body of the liquor [6]. Polyphenols have an important effect on the color and bitterness of the wine. Furthermore, it has been shown to be a significant contributor to antioxidant activity and plays an important role in the prevention of cardiovascular disease [7]. Furthermore, the color and aroma of red wine are two of the most important quality indicators, which largely determine the quality of wine [8]. Nowadays, more innovative and complex wines are considered to possess higher quality.

Therefore, it is very important to study the technology and application of wine brewing to improve the complexity and quality of wine. Polyphenols and volatile compounds, along with their interactions, are responsible for color and aroma, respectively [9]. The content of these compounds can be achieved by blending fermentation [10]. Previous research found that when different varieties of grapes are blended for maceration and fermentation, some grapes in the blend must may profit from extra molecules offered by other varieties, causing more complex structures than single-varietal wines [1,11,12]. This type of wine can achieve more complex aroma characteristics and better color stability. Color stability is directly related to the quality of wine [8]. Studies by some authors suggest that the co-pigmentation of anthocyanins forms the first step toward a more stable polymeric pigment. Molecular binding between anthocyanins or with other molecules called cofactors can occur. These associations result in a hydrophobic site involving more anthocyanins participating in the wine color and improving the quality of wine [5,11,13].

Some grape varieties may be rich in certain cofactors, while others may be deficient. Furthermore, not all grapes contain the same number of anthocyanins and polyphenols, so some varieties may benefit from the presence of other varieties that may contain an excess of these compounds. In addition, Escudero Gilete et al. demonstrate that blended wines from different grapes provide a more equilibrated ratio of anthocyanins/flavanols [14], which means that mixing different cultivars of wine can implement better flavor balance.

The Cabernet Sauvignon grape is in a dominant position in the market because of its easy planting, high tannin, deep color, abundant style and a strong sense of structure [13]. However, due to the use of similar strains, fermentation processes, fermentation equipment, etc. [15], Cabernet Sauvignon wine presents a single style and serious homogenization. Therefore, the selection of local fermentation strains and the development of a unique fermentation process to improve the quality of wine has become a hot research and development area [16]. We selected four monovarietal wines of different chromatic, phenolic and aroma properties, namely, Merlot (ML), Marselan (MS), Syrah (SY) and Point Noir (PN), and used high-performance liquid chromatography (HPLC) and headspace solid-phase microextraction with gas chromatography coupled to tandem mass spectrometry (HS-SPME-GC-MS/MS) to determine the effects of different varieties and various blending methods on the physicochemical indicators, nutrients, color and flavor of Cabernet Sauvignon wine [17]. The results will provide a theoretical basis for the flavor improvement of Cabernet Sauvignon wine.

## 2. Materials and Methods

### 2.1. Winemaking and Samples

The wine grapes (Cabernet Sauvignon, Merlot, Marselan, Syrah and Point Noir) used in this study were harvested from the Manas production area of Xinjiang in China on 3 September 2021 at optimum maturity. The physicochemical indexes of different grape varieties are shown in Table S1. Two types of blending methods were used in this study. Co-grapes (CG-treated): mixed grape musts before fermentation were prepared by using Cabernet Sauvignon as the base and Merlot (ML), Marselan (MS), Syran (SY) and Point Noir (PN) as modifiers in volumetric proportions of 20% (CGM (CS:ML = 80:20), CGS (CS:MS = 80:20), CGY (CS:SY = 80:20), CGP (CS:PN = 80:20)). Co-wines (CW-treated): mixed wines after fermentation were prepared by using Cabernet Sauvignon wine (CSW) as the base and Merlot (ML), Marselan (MS), Syran (SY) and Point Noir (PN) wine as modifiers, respectively, in volumetric proportions of 20% (CWM (CS:ML = 80:20), CWS (CS:MS = 80:20), CWY (CS:SY = 80:20), CWP (CS:PN = 80:20)).

All wine samples were separately fermented at the same condition (as follows). CS, ML, MS, SY, PN, CGM, CGS, CGY and CGP musts were crushed and collected into 50 L stainless-steel containers. Then, 60 mg/L of SO_2_ and 50 mg/L of pectinase were added to the musts [13]. After being macerated for 28 h, 200 mg/L of dried active yeast (Fermol Rouge AEB, Brescia, Italy) was added to the musts, in accordance with commercial specifications. Alcoholic fermentation was carried out at 25 °C for 7–10 days to dryness (residual sugar less than 4 g/L) and stopped by separating pomace and adding 50 mg/L of SO_2_ [17]. The wine samples were stored at 10 °C until further analysis. All experiments were repeated in triplicates.

### 2.2. Enological Parameters

The soluble solids content was measured by a digital refractometer (Atago PR-101R, Tokyo, Japan). The pH of the wines was monitored by a digital pH meter (pHS-3C, Shanghai Aohaosi Instrument, Shanghai, China). Titratable acidity, alcohol and free SO_2_ in wine samples were tested according to the methods of the International Organization of Vine and Wine [18], Residual sugar content was assessed using the dinitrosalicylic acid (DNS) method and the volatile acidity was determined following the method proposed by Miller, L [19].

The organic acids in wine samples were based on a previous method with some modifications [6]. Each sample was centrifuged and filtered through a 0.22 μm nylon membrane, and organic acids were identified by HPLC equipped with a Spursil C18 column (250 mm × 4.6 mm × 5 μm, Dima Technologies, Shanghai, China). The column oven temperature was maintained at 20 °C. The injected sample volume was 10 µL, while the mobile phase was 0.1% (*v*/*v*) formic acid at a flow rate of 1 mL/min.

### 2.3. Determination of Phenolic Compounds

Total phenolic content of wine samples was determined by a spectrophotometer according to the Folin-Ciocalteu colorimetric method [20]. The total flavonoid content was determined according to the method of Peinado et al. [21]. Oligo proanthocyanidins (OPC) content was determined using the vanillin-hydrochloric acid colorimetric method [22]. The total monomeric anthocyanins (TMA) content was evaluated by the pH differential method [23].

HPLC analysis was performed based on the methods of Alejandro, Cáceres-Mella et al. with some revisions [7]. Wines were analyzed in a Hewlett-Packard 1100 chromatographic system equipped with a quaternary pump, a UV-vis detector, an automatic injector, and Chemstation software (Empower 3). A Dikma C18 column (5 µm, 250 × 4.6 mm, Diamonsil Plus Technology, Shanghai, China) was used to separate the phenolics. Water-acetic acid (10:90) as solvent A and methanol as solvent B were used. The elution profile was as follows: 0–20 min 95% A-5% B; 20–35 min 75% A-25% B; 35–55 min 60% A-40% B; 55–60 min 95% A-5% B; the flow rate was 0.4 mL/min and the injection volume was 20 µL.

Phenolic compounds were identified by comparing retention times and chromatographic spectra of samples with gallic acid (y = 27200x − 47500, R^2^ = 0.9982), catechin (y = 7206x − 24900, R^2^ = 0.9989), epicatechin (y = 8870x − 3320, R^2^ = 0.9989), rutin (y = 4310x − 4130, R^2^ = 0.9999), coumaric acid (y = 16400x − 2980, R^2^ = 0.9992), procyanidin B1 (y = 6290x − 15900, R^2^ = 0.9979), chlorogenic acid (y = 16000x − 43700, R^2^ = 0.9963), vanillic acid (y = 17900x + 16700, R^2^ = 0.9969), ferulic acid (y = 33000x − 9086, R^2^ = 0.9994), quercetin (y = 18200x − 51100, R^2^ = 0.9986) and kaempferol (y = 18600x − 52000, R^2^ = 0.9951) standards, and phenolic compounds were quantified using a standard calibration curve. All qualitative and quantitative analyses of the phenolic composition were performed in triplicate.

### 2.4. Determination of Antioxidant Activity

The DPPH radical scavenging activity of samples was determined according to the method of Gadow et al. [24]. The free scavenging ability of ABTS+ was performed following a modification of the technique of Re et al. [25]. The CUPRAC reduction forces of the samples were analyzed by the Apak method [26]. The FRAP-reducing power assays of the sample were determined by Wei et al. [27].

### 2.5. Colour Evaluation of Wines

The wine color was measured using the method of Ayala et al. [28]. Samples were centrifuged at 3500 rcf, and the supernatants were filtered through a 0.45 μm nylon membrane prior to the color determination. The absorbance spectrum of the wine (380–770 nm) was recorded in a 1 mm-width quartz cuvette using a spectrophotometer. Reference blank measurements were made with the cuvette filled with distilled water.

### 2.6. Analysis of Volatile Compounds

Wine volatile composition was determined by headspace solid phase microextraction-gas chromatography-mass spectrometry (SPME-GC-MS) according to previous reports with some modifications to the temperature program [29].

The GC-MS system was an Agilent 7890B GC incorporated with an Agilent 5977 mass spectrometer (Agilent Technologies Inc., Palo Alto, CA, USA). The column used was a 30 m × 0.25 mm HP-INNOWAX capillary with 0.25 μm film thickness (Shanghai Shimadzu Laboratory Equipment Co., Shanghai, China). The carrier gas was helium, and the flow rate was 1 mL/min. The temperature program was as follows: 40 °C for 5 min, 50 to 86 °C at 3 °C/min, 86 to 90 °C at 0.8 °C/min, 90 to 180 °C at 2.5 °C/min, 180 °C for 3 min, then at 25 °C/min, up to 230 °C, with a final holding time of 5 min.

The mass spectra of the sample volatile compounds were compared with those in the NIST library, and the volatile aromatic compounds were identified by comparing the retention time and mass spectra of the sample volatile compounds with the standard display [5]. Utilizing the odor activity value (OAV), each volatile compound’s unique contribution to the aroma of the wine was assessed. OAV is calculated by dividing the concentration of each volatile substance in wine by its perception threshold [30].

### 2.7. Sensory Analysis

Wine sensory analysis was conducted according to Peng et al. [31]. The well-trained panelists (18 ladies and 12 males) examined the aroma characteristics of the samples. The team members were required to conduct a consensus on terminology and describe sensory odor. The description of sensory odor includes bitterness, astringency, viscosity, mouthfeel, fruity, floral, and herbal, and the score from 0 to 20 represents the sensory odor from weak to strong.

### 2.8. Statistical Analysis

The one-way analysis of variance (ANOVA) with a Duncan test of the SPSS 19.0 software was used to examine the variation between samples (SPSS Inc., Chicago, IL, USA). The effects of different samples of the aroma profiles were evaluated by principal component analysis (PCA) using SIMCA 14.1 (Umetrics, Malmö, Sweden). Origin (2021) was used to generate histograms, radar charts and overlays. For each sample group, three parallel samples were analyzed.

## 3. Results and Discussion

### 3.1. Analysis of Physicochemical Characteristics

Table 1 shows the chemical composition of all wine samples according to the different grape varieties (Cabernet Sauvignon, Merlot, Marselan, Syrah and Point Noir), and the use of different blending methods (Co-grapes and Co-wines). The total acid of all wines ranged from 4.96 to 6.8 g/L, and that peaked for CGP wines treated with co-grapes. In addition, a high level of total acids contributes to the microbial stability and the freshness of these wines [13,32]. The alcohol content ranges from 10.23 to 12.89%, of which ML has a maximum alcohol content of 12.89%. Volatile acidity, with an overall average of 0.39 ± 0.1 g/L, had no effect on wine quality, and the presence of free SO_2_ in wine (13–29 mg/L) was sufficient to protect wine from further oxidation and microbial contamination [15]. Of note was that the volatile acidity (<1.2 g/L) and the SO_2_ (<100 mg/L) content were under the legal limit established for wines [16,18].

### 3.2. Cluster Analysis of Organic Acids

**Figure 1:**
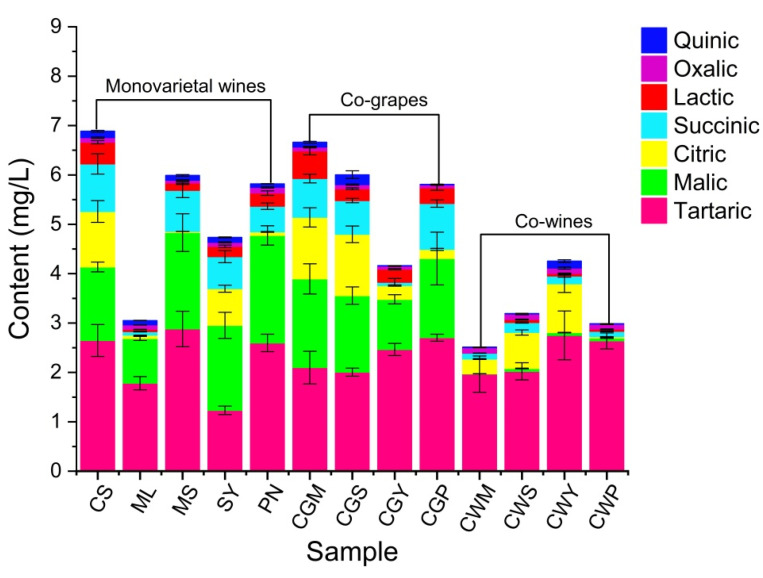
The organic acid content of wine samples from different varieties and blending methods. The monovarietal wine: Cabernet Sauvignon (CS), Merlot (ML), Marselan (MS), Syran (SY) and Point Noir (PN); (CGM (CS:ML = 80:20), CGS (CS:MS = 80:20), CGY (CS:SY = 80:20), CGP (CS:PN = 80:20)) by co-grape treatment; CLM1 (CS:ML = 90:10), CLM2 (CS:ML = 80:20), CLM3 (CS:ML = 70:30) by co-wines treatment.

The organic acids have a great influence on the flavor balance of the wine, as well as on the chemical stability and pH, thus affecting the quality of wine [30]. In addition, organic acids may have protective effects against various diseases due to their antioxidant activity [33]. A total of five organic acids (Figure 1) were identified in wine samples: tartaric, malic, citric, succinic, lactic, oxalic and quinic. Tartaric acid was the predominant acid in wines, with concentrations ranging from 1.23 to 2.88 g/L, followed by malic acid. The sum of tartaric and malic acids can represent more than 60% of the total amount of acid in wines. Malic acid provides a sharp and strong taste sensation, while excessively high levels of malic acid can cause a pungent taste [34].

Regarding the varietal factor, it can be summarized that the MS and PN wines showed higher tartaric and malic acids than the other wine samples. Citric acid slows down yeast growth and participates in biochemical and metabolic processes [17], up to 1.25 g/L in CGM and CGS wines. Lactic acid improves the nutritional value and the stability of wine, reduces sharp flavors, and increases the complexity of wine (baking, butter, vanilla, spice and smoke) and fruit flavors [34]. The highest lactic acid content was found in CS wine, while no lactic acid content was detected in CWM wine. In the co-wine treatment, the content of lactic acid was only one-tenth that of the co-grape treatment. In general, the contents of malic acid and lactic acid in co-wine treatment were significantly lower than those in co-grape treatment, which may be related to the phase reaction between compounds and microbial metabolism during co-grape processing [33].

### 3.3. Analysis of Total Phenolic, Total Flavonoid, Total Anthocyanin and Proanthocyadin

**Figure 2:**
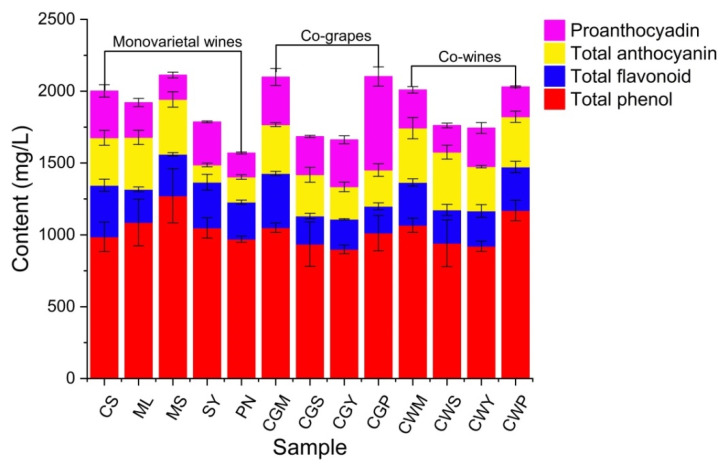
The composition of total phenol, total flavonoid, total anthocyanin and proanthocyadin of wine samples blended with different varieties and blending methods.

Phenolic compounds are involved in fermentation, prevent microbial spoilage, provide aroma to fermented juice, and control fermentation rate [34]. In addition, the high polyphenol content of berries makes them an important source of dietary antioxidants, reducing the risk of chronic diseases, including cardiovascular disease and cancer [35]. The content of polyphenols in different varieties of wine was significantly different (*p* < 0.05). Before fermentation, MS wine had the highest total phenol concentration of 1272 mg/L (Figure 2). After blending, the total phenolic content in CWP can reach up to 1169.2 mg/L. The reduction in total phenolic amount is due to the oxidation of phenolic compounds after fermentation and the combination of hydroxybenzoic acid compounds with alcohol and tannin during fermentation [13]. This may also be related to yeast metabolism in fermentation broth [34]. Higher levels of total flavonoids and total anthocyanins were found in co-wines, reaching 231.5–303.7 and 349.3–402.2 mg/L, respectively.

### 3.4. Composition of Phenolic Compounds

**Figure 3:**
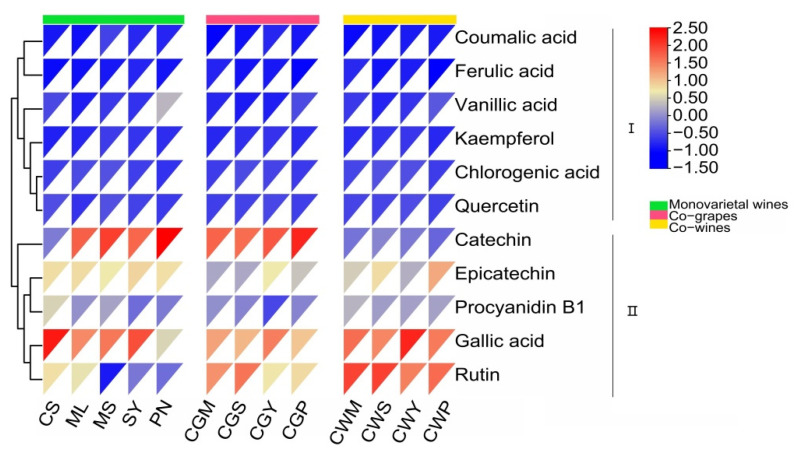
Monomeric phenol content of wine samples (mg/L) from different varieties and blending methods.

Eleven phenolic compounds, including gallic acid, catechin, epicatechin, rutin, coumalic acid, procyanidin B1, chlorogenic acid, vanillic acid, ferulic acid, quercetin and kaempferol were identified in the wine samples (Figure 3). Gallic acid was the dominant phenolic compound in all wines, ranging from 16.6 to 54.3 mg/L, and its main sources were grape seeds and oak cooperage [12,36], accounting for 30 to 50% of the total phenolic compounds. Among the wines, SY had the highest level of gallic acid (54.3 mg/L), in which content was approximately two to three times more than those of others. Followed by catechin, its concentration ranged from 6.59 to 50.71 mg/L. Much of the research on the health benefits of red wine focused on catechin content, which has antioxidant properties and is a polyphenolic antioxidant plant metabolite, accounting for 30–50% of phenolics [9,33,37]. The catechin content in co-grapes was three times that in co-wine treatment, which may be due to the impregnation effect in the early stage of co-grape treatment. It is worth noting that higher rutin content was found in the co-wine treatment, while previous studies have found no record of this phenomenon.

### 3.5. Antioxidant Activity

**Figure 4:**
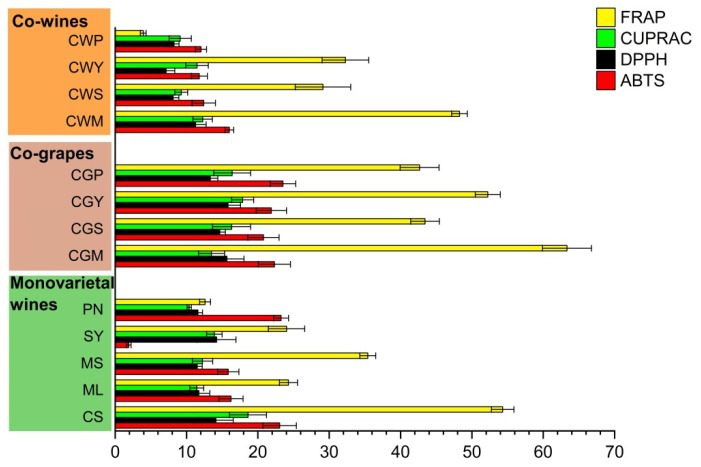
The antioxidant properties (mM TEs/L) of wine samples with different varieties and blending methods.

Antioxidants in wine can be used to prevent coronary disease and atherosclerosis, which is beneficial to human health [22]. The antioxidant activity of wine samples was evaluated by four different methods (ABTS, DPPH, CUPRAC and FRAP) [38]. Among all the samples, the antioxidant activities were significantly different. When compared to the single wine, all blending wines showed important activities (Figure 4).

Among the wines from the five grape varieties, the antioxidant capacity of CS was the strongest, followed by those of ML and MS. These findings are in line with the research by You et al., who found the Cabernet Sauvignon wines not only had higher horizontal phenolic antioxidants, for instance, catechin, gallic acid and epicatechin, but also higher antioxidant capacities compared to Merlot wines [37]. Within co-grapes, CWS wines showed the highest antioxidant activity based on ABTS, DPPH, and the FREP assays.

For co-wines, the highest antioxidant activity values were observed in the CWM, while the CWP wines yielded the lowest results. Overall, co-grapes exhibited higher antioxidant properties compared to co-wine, which may be related to the impregnation in the early stage of co-grape treatment. This conclusion was supported by Anli et al., who found that the red wines from the Syrah and Grenache varieties had higher antioxidant activity after prolonged maceration and were responsible for the relative inhibition of LDL oxidation by 60% in humans [36].

### 3.6. Analysis of the Antioxidant Capacity of Phenolic Compounds

**Figure 5:**
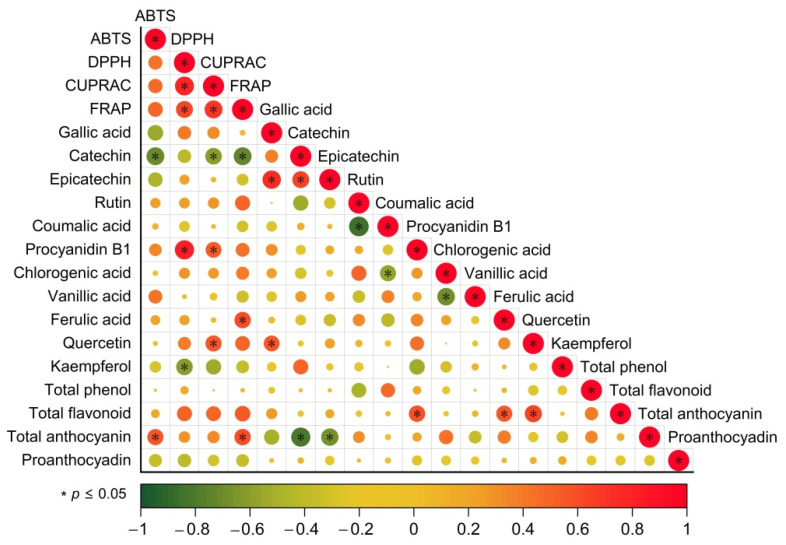
The correlation between phenolic compounds and antioxidant activity. Strong and weak correlations are indicated by the large and small circles, respectively. The color of the scale bar denotes the nature of the correlation; 1 indicates a perfect positive correlation (red) and -1 indicates a perfect negative correlation (green). Significant correlations *p* ≤ 0.05) is indicated by *.

The health-protective properties of wines are attributed to their antioxidant activity, which is their ability to eliminate free radicals [39]. Wines were assessed for concentrations of phenols, flavonoids, anthocyanins, ABTS, DPPH, CUPRAC and FRAP. Correlations among these chemical parameters were investigated using SIMCA software (Figure 5). Significant correlations among different antioxidant assays (DPPH, ABTS, CUPRAC and FRAP) were found in blending wine samples.

Similarly to the previous assays, anthocyanins have been shown to be very effective antioxidants in red grape extracts and various red wines [38,39]. As shown in Figure 5, anthocyanin content has a good correlation with the antioxidant activity of red wine. There is a strong correlation between total flavonoids and DPPH, CUPRAC and FRAP antioxidant activities [40]. In addition, Anli et al. found a significant correlation between total phenols and antioxidants, but no similar results were found in this study [36].

In terms of monomeric phenols, gallic acid, epicatechin, quercetin and kaempferol were found to be highly correlated with antioxidant activity in wine. Earlier, the high antioxidant activity of these compounds was confirmed by several studies [3,38]. According to the chemical structural characteristics of these phenolic compounds, the strong antioxidant activities of catechin, epicatechin and quercetin may be due to the fact that their B-rings have catechol-type structures as reducing agents.

### 3.7. Analysis of Wine Colour

**Figure 6:**
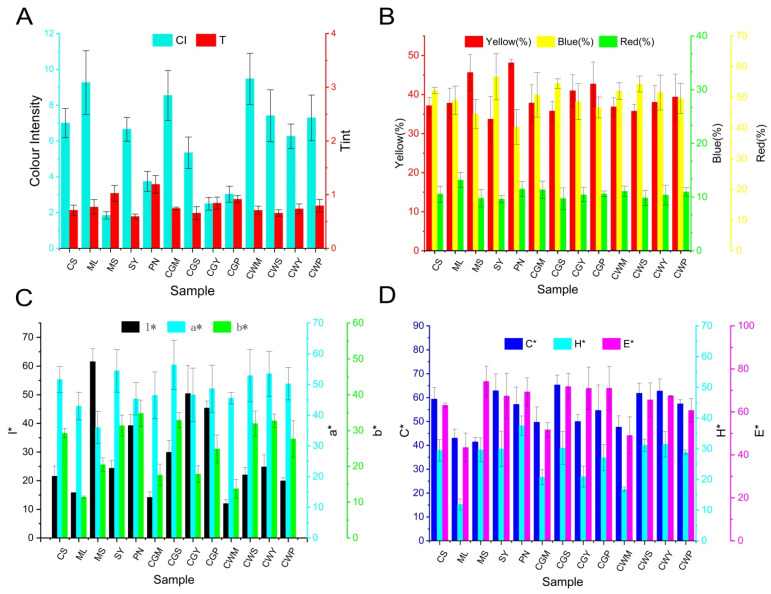
The analysis of colorimetric indices in wine samples: (**A**) Colour intensity (CI) and tint (T); (**B**) %Red; %Yellow and %Blue; (**C**) l*, a*, b*; (**D**) C*, H*, E*. The * is part of the full name to distinguish it from the letters l, a, b, C, H, E.

Figure 6 illustrates the effect of blending treatments on wine color in terms of the Color Index (CI, %Red, %Blue, %Yellow and Hue) and CIELAB variables (l*, C*, H*, a* and b*). The color parameters of the blended wines of different varieties are significantly different. CGS can improve the red tint, and CGP and CGM increase the blue hue. The different effects of blending groups on color quality improvement may depend on the proportion of phenolic content.

CGM showed the highest chroma values, while as for hue, all wines showed similar results. In addition, compared with co-wines, the co-grapes treatment showed higher red parameter values and lower yellow parameter values, which may be related to the impregnation effect of the mixed grape treatment in the early stage. This was similar to the study of Gomez-Miguez and Ortega-Heras et al., who found that impregnation had a positive effect on the color value of wine [41,42].

As shown in Figure 6D, the co-grape treatment showed higher comprehensive color (E*) than co-wines. This conclusion was consistent with the research of Monagas et al. [12], and they found that when blending, even if only 10% of the modifier wine is added, it is possible to perceive the color difference due to the blending of the wine, making the blended wine more chromatic and darker.

### 3.8. Aromatic Volatile Composition and Content

Wine aroma is a very important sensory parameter [43,44]. In wine, the aroma is affected by many different factors, such as grape varieties, climate, fermentation conditions, and other winemaking microbial flora and production technology [1]. The resulting aromatic characteristics play a key role in the characterization of typical wines [43].

**Figure 7:**
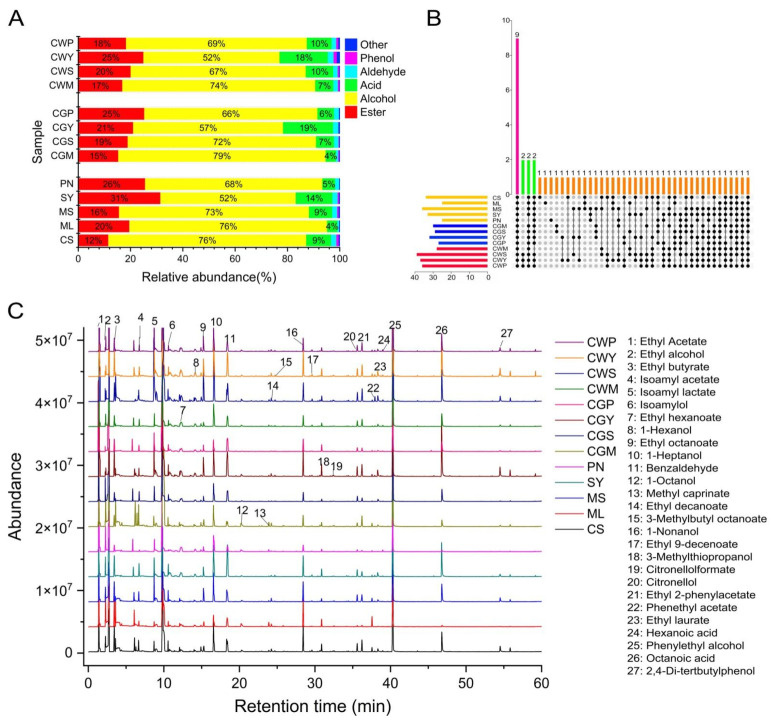
The composition of volatile compounds in wines (**A**). The upset diagram of the wine samples (**B**). Black dots indicate the common aroma compounds in the sample. Different colors represent different treatments and a common number of aroma compounds. The total ion chromatogram of wine samples (**C**).

This study identified 53 different aromatic compounds, grouped by functional group, including 23 esters, 12 alcohols, 8 acids, 4 aldehydes, 1 phenol, and 5 other compounds. The results showed that there were obvious differences between some blends and their parents. The identified volatile compounds, classified by chemical types, were shown in Table S3. The percentage content of total alcohols, esters, aldehydes, fatty acids, phenol, and others are in Figure 7A.

The most abundant compounds are alcohol, which contributes about 57–76% of the total volatiles, followed by esters (12–31%). The total alcohol content of CS and ML was significantly higher in all wines than the others, and the co-grape treatment had a higher total alcohol content compared to the co-wines [15]. The upset diagram of the wine samples is in Figure 7B. Furthermore, a total chromatogram of wine samples is shown in Figure 7C.

#### 3.8.1. Alcohol

Alcohol may be derived from yeast fermentation. C-6 alcohol is a kind of common plant volatile with a typical “green” odor [2]. They are formed during grape maceration [44,45]. These are correlated to the woody parts of the leaves in wine. They are derived from membrane lipids via the lipoxygenase pathway [46]. The quantity of hexanol was distinctly different in all wines (*p* < 0.05). The content of 1-hexanol in CY wine is the highest, reaching 85.73 mg/L, followed by CS wine, 51.05 mg/L. The blending treatment reduces the *n*-hexanol content to two to three times the original, which suggests that blending results in a significant reduction in the green character of the wine [12,15]. In addition, the high content of short-chain alcohols, also called fusel, will adversely affect the aroma of the wine. Higher alcohols are mainly formed by yeast metabolism during alcoholic fermentation [45]. These compounds can be attributed to their strongly pungent odor and herbal taste [29].

Wines blended with Pinot Noir and Cabernet Sauvignon grapes had significantly the highest concentrations of benzyl alcohol and *n*-octanol compared with other wines, with pleasant rosy, citrus aromas. Additionally, the highest levels of citronellol, 6-Nonen-1-ol and 3-Methylthiopropanol were found in blends of Cabernet Sauvignon and Syrah varieties. In the blended wine, it was observed that the content of higher alcohol was significantly affected by the blending method (*p* < 0.05). Compared with the co-wine treatment, the content of 1-heptanol, phenylethyl alcohol, isoamylol and ethyl alcohol was significantly higher in the co-grape treatment, which may be related to the maceration effect in the early stage of the co-grape treatment, which is consistent with the research of Sánchez-Palomo, who found that the concentration of higher alcohol is significantly correlated with immersion time [45].

#### 3.8.2. Ester

**Figure 8:**
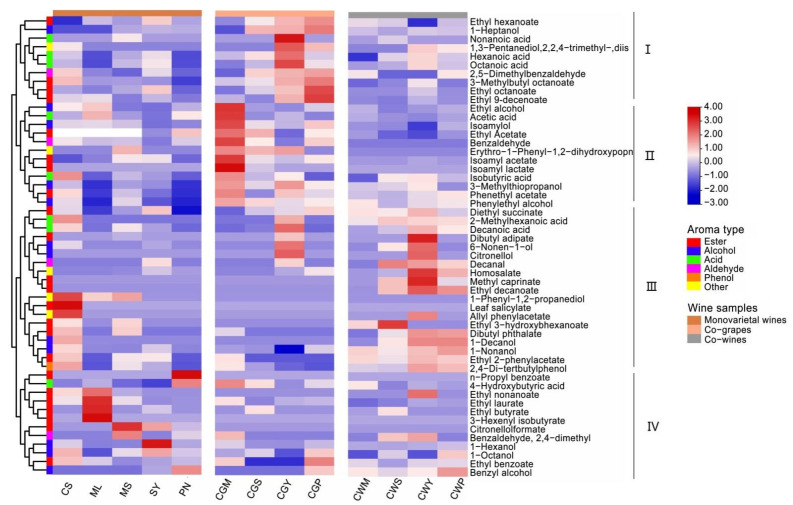
Heatmap cluster analysis of wine samples with different varieties and blending methods.

Esters have long been considered an important contributor to wine aroma as they are the main source of fruity aromas [1]. Most esters found in alcoholic beverages are secondary metabolites produced by yeast during alcoholic fermentation [47]. The production of different esters in wine depends on many winemaking factors such as temperature, the type of yeast used for fermentation and ventilation, etc. [47]. Twenty-three types of esters were tested in this study, and they were evidently different in the types and contents of all samples (Figure 8). The study found a significantly higher amount of ethyl acetate, isoamyl acetate, isoamyl lactate, phenethyl acetate and ethyl laurate, while higher levels of ethyl hexanoate, ethyl octanoate, 3-methylbutyl octanoate, ethyl benzoate and ethyl 9-decenoate were found in CGP wines. As far as the blending method is concerned, the content of ethyl acetate, isoamyl acetate, isoamyl lactate, ethyl octanoate, ethyl hexanoate, 3-methylbutyl octanoate and ethyl 9-decenoate in the co-grapes treatment is significantly higher than that in the co-wines treatment, which indicates that the mixed grape treatment increased the content of esters [31].

#### 3.8.3. Acid

The production of fatty acids is related to the initial composition of the must and fermentation conditions. These compounds have been described as fruity, fatty cheese, and rancid notes [46]. Volatile fatty acids are of great benefit to the complexity of wine aromas even when present at subsensory threshold levels, and they negatively affect wine aroma when they exceed their thresholds [16]. As shown in Figure 8, higher concentrations of nonanoic acid and acetic acid were found in CS wine, which reached 119.8 and 112.3 mg/L, respectively. In terms of variety, Cabernet Sauvignon blended with Merlot showed a significant increase in butyric and acetic acid. The co-grapes treatment increased the concentration of octanoic acid in the wine, while the co-wines treatment increased decanoic acid in the wine, and the results of the blending wine were the same as Lorenzo et al., who found that blending can increase caprylic and capric acid content in wines [5].

### 3.9. The Analyses of Principal Component

Principal Component Analysis (PCA) was used to assess the effect of parameters (different blended varieties and blending methods) on the aroma profile of the analyzed wines [4]. The first two principal components, PC1 and PC2, accounted for 59.9% of the total variance.

**Figure 9:**
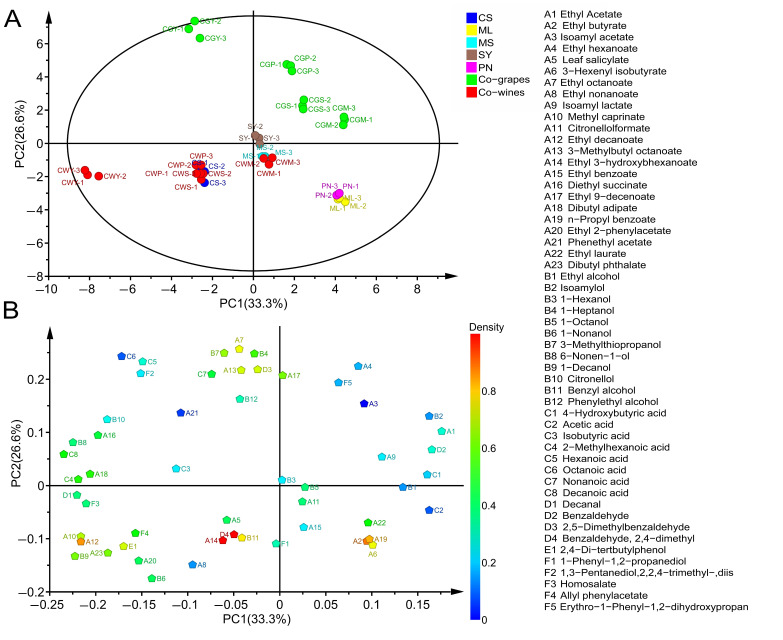
Principal component analysis (PCA) (**A**) and the loadings plot (**B**) of volatile compounds. Figure (**A**) includes all sample names. The serial numbers A1 to F5 in the chart on the right delegate the volatile compound names in Figure (**B**), respectively.

The projection of the wine samples on PC1 showed a clear separation according to the grape variety (Figure 9A). Therefore, wines were mainly divided into three groups: PN and ML were one group (obviously separated from other wines, in the positive half axis of PC1), SY and MS formed a second group near the origin, and the third group was composed of CS wines on the negative part of PC1. As shown in Figure 9A, in terms of varieties, CS and other wine aroma compounds show a clear separation, which is similar to the research of Jiang et al., and they found that CS wines were significantly lacking in fruit aroma compared with other varieties [16]. The composition of the different varieties of wines was determined and presented in the scatter plot in Figure 9B. Therefore, the main components of wine separation were ethyl laurate, ethyl butyrate, *n*-propyl benzoate and 3-hexenyl isobutyrate. They were negatively related to PC1 and were specific for PN and ML.

Regarding PC2, further differences in wine samples were related to the method of blending. Therefore, as shown in Figure 9A, the co-grapes treatment was placed in the positive part of PC2, while the co-wines treatment was placed in the negative part of PC2, which implied that PC2 was correlated with the blending methods. The main ingredients that cause differences in wines from different practices are ethyl octanoate, 1-heptanol, ethyl 9-decenoate ethyl hexanoate, isoamyl acetate, isoamyl lactate, 3-methylthiopropanol and 3-methylbutyl octanoate, especially suitable for co-grape wines.

### 3.10. The Effect of Blending on Aroma Characteristics

**Figure 10:**
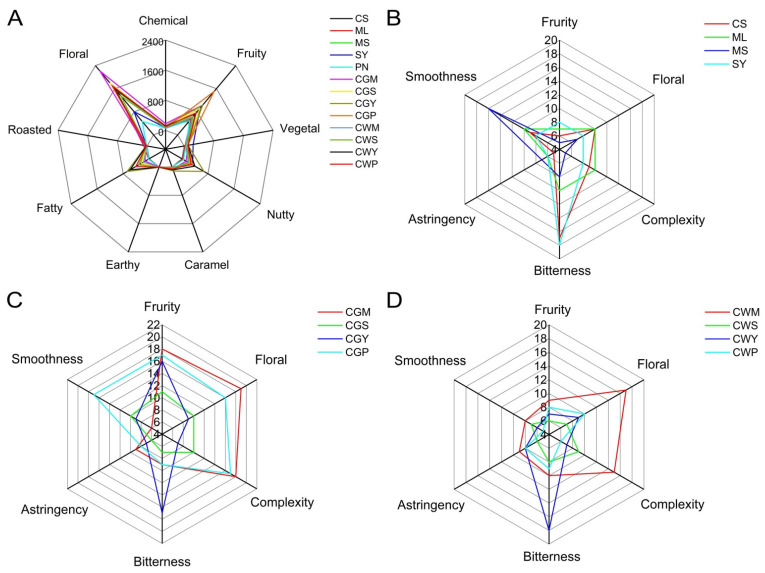
Description of aroma series of wine samples (**A**). The sensory analysis of Monovarietal wines (**B**). The sensory analysis of wine samples with co-grapes treatment (**C**). The sensory analysis of wine samples with co-wines treatment (**D**).

Of the 53 compounds analyzed and quantified, only 9 had OAVs greater than or equal to 1.00 in wine (Figure S1). The intensity patterns of the aroma series showed that the aroma of wines in this study were mainly composed of fruity, floral, nutty and fatty aromas (Table S2). In addition, the caramel range also contributes to the wine’s overall aroma (Figure 10A).

The floral series is the most intense main aroma series of all treatments. The main contributors to this series are leaf salicylate, citronellolformate, phenethyl acetate, ethyl laurate, 1-octanol and 1-decanol. The effect of different varietal blends on this aroma family is markedly different. Leaf salicylate, citronellolformate and phenethyl acetate reached the highest levels in CGW wines. It is worth noting that the concentration of floral series in the blended wine samples is distinguished higher than the blended wine.

The fruit series is another main aroma series in this study, dominated by ethyl octanoate, ethyl hexanoate, ethyl laurate, ethyl 9-decenoate, isoamyl acetate and phenethyl acetate. The highest concentration of fruit aromas was found in CGP wine samples, followed by CGW wines. Similarly, the concentration of fruit series in the blended wine samples is about twice that of the blended wine.

Fats and nuts were the other two main aroma families in this study. The fats series was dominated by five compounds (ethyl nonanoate, ethyl decanoate, 1-decanol, isobutyric acid and octanoic acid), and the nuts series was dominated by four compounds (benzyl alcohol, octanoic acid and benzaldehyde). The highest fat and nut concentration was found in CGY wines, followed by CWY. Interestingly, the fat series by the co-wines treatment is significantly higher than co-grapes.

### 3.11. Sensory Analysis of Wine

The results of the sensorial analysis are shown in Figure 10B–D. The co-grape wines in CGP, CGM and CGY showed hints of fruity aromas (Figure 10C). This note was especially high in co-grapes than in co-wines. The wines made between crosses from Merlot and Cabernet Sauvignon are well-balanced aromatically, according to the tasters, with some floral notes [16]. In general, the results also show that the CGM, CWM and CGP wines were positively appreciated by the tasters, with higher “floral” and “fruity” scores compared with the other wines, which is in accordance with the higher ethyl cinnamate content of these wines.

The bitterness of CS and SY was higher than other wines (Figure 10B), but there was no significant change in bitterness in CGY and CWY wines after mixing, which indicated that the blending did not cause a significant change in bitterness. Higher astringency in MS and CWP wines may be related to higher total phenolic and flavonoid content in wines [40]. In general, CGM and CGP wines have the smoothest taste compared to other wines.

## 4. Conclusions

This work provides a better knowledge of the nutritional, taste, color and volatile composition of Cabernet Sauvignon wines elaborated by the blending technique. The results showed that the wines brewed by the blending technique present a more complex chemical characteristic and aroma complexity than monovarietal wines. High total phenols, total flavonoids and antioxidant activity were found in CGM, CGP and CWP wines. In terms of color parameters, CGM and CGS have a higher red tint, and CGM has a higher blue tint. These wines show better colors. In addition, the wines made by the CGP combination performed best in the fruity characteristic due to its high ester concentrations. The CGY and CWY wines had a high content of ester, alcohol and aldehyde compounds, showing the characteristics of nutty and fatty aromas. Notably, the co-grape treatment had higher antioxidant properties and aroma complexity compared to the co-wine treatment. Our research provides a feasible alternative to traditional methods to improve the chemical and organoleptic characteristics of Cabernet Sauvignon wines.

## Figures and Tables

**Table 1:** Physicochemical indexes of wine sample.

Wine Sample	Residual Sugar (g/L)	Total Acid (g/L)	Volatile Acid (mg/L)	Sulfur Dioxide (mg/L)	pH	Alcohol %(*v*/*v*)
CS	3.60 ± 0.35 ^ab^	6.32 ± 0.99 ^abc^	0.62 ± 0.01 ^c^	29.45 ± 1.91 ^cd^	3.90 ± 0.31 ^a^	11.18 ± 1.82 ^ab^
ML	2.66 ± 0.38 ^cde^	6.32 ± 1.03 ^abc^	0.36 ± 0.05 ^d^	20.43 ± 2.46 ^e^	4.23 ± 0.34 ^a^	12.89 ± 1.92 ^a^
MS	1.98 ± 0.07 ^e^	6.32 ± 0.81 ^abc^	0.97 ± 0.09 ^a^	29.56 ± 4.58 ^bc^	3.43 ± 0.31 ^a^	11.60 ± 0.94 ^ab^
SY	1.81 ± 0.26 ^e^	6.05 ± 0.35 ^abc^	0.24 ± 0.02 ^f^	33.78 ± 5.77 ^ab^	3.95 ± 0.57 ^a^	10.70 ± 0.64 ^b^
PN	3.80 ± 0.63 ^ab^	6.45 ± 0.42 ^ab^	0.62 ± 0.02 ^bc^	36.32 ± 4.21 ^a^	4.33 ± 0.46 ^a^	12.76 ± 0.79 ^ab^
CGM	4.00 ± 0.56 ^a^	6.05 ± 0.67 ^abc^	0.72 ± 0.13 ^b^	29.45 ± 2.91 ^bc^	4.09 ± 0.46 ^a^	12.82 ± 0.39 ^ab^
CGS	2.32 ± 0.37 ^de^	4.95 ± 0.75 ^bc^	0.23 ± 0.02 ^ef^	17.58 ± 2.27 ^ef^	3.73 ± 0.52 ^a^	11.34 ± 1.84 ^ab^
CGY	2.41 ± 0.34 ^de^	6.55 ± 1.01 ^ab^	0.33 ± 0.03 ^de^	27.37 ± 2.93 ^bc^	3.94 ± 0.43 ^a^	10.71 ± 2.2 ^ab^
CGP	1.98 ± 0.08 ^de^	6.85 ± 0.92 ^a^	0.03 ± 0.00 ^g^	13.28 ± 1.98 ^f^	4.11 ± 0.68 ^a^	12.88 ± 1.12 ^a^
CWM	3.20 ± 0.44 ^c^	5.10 ± 0.86 ^bc^	0.23 ± 0.03 ^def^	28.93 ± 3.45 ^bc^	3.97 ± 0.08 ^a^	12.42 ± 1.31 ^ab^
CWS	2.32 ± 0.35 ^de^	4.75 ± 0.85 ^c^	0.33 ± 0.02 ^def^	25.69 ± 3.45 ^cd^	3.78 ± 0.61 ^a^	10.53 ± 1.11 ^ab^
CWY	2.49 ± 0.05 ^cd^	5.35 ± 0.91 ^abc^	0.25 ± 0.03 ^def^	27.72 ± 3.17 ^c^	3.91 ± 0.49 ^a^	11.46 ± 0.96 ^ab^
CWP	3.90 ± 0.67 ^ab^	5.80 ± 0.83 ^abc^	0.21 ± 0.04 ^ef^	26.32 ± 3.77 ^cd^	3.98 ± 0.51 ^a^	10.23 ± 1.33 ^ab^

^a^ Values are expressed as mean ± standard deviation (*n* = 3). Different superscripts (a, b, c, d, e, f, g) in a column denote significant differences among samples (*p* < 0.05, Tukey’s HSD test).

## Data Availability

Data are contained in the article.

## Supplementary Materials

The following supporting information can be downloaded at: https://www.mdpi.com/article/10.3390/foods11213332/s1, Figure S1. The key volatile compounds in different wine samples (OAV > 1 ); Table S1. Physicochemical indexes of different grape varieties; Table S2. Volatile compounds identified in this work and their aroma parameters; Table S3. Concentration (µg/L, mean ± SD) of the volatile compounds in different blending treatment wines.
