# Supplementary material for: Effects of Blending on Phenolic, Colour, Antioxidant and Aroma Components of Cabernet Sauvignon Wine from Xinjiang (China)
Source: Foods. 2022 Oct 24;11(21):3332. doi: 10.3390/foods11213332 (PMC9653794; doi:10.3390/foods11213332)
Supplement: Supplementary file 1 [file foods-11-03332-s001.zip › foods-1950785-supplementary.pdf]

“Supporting Information”

Effects of blending on phenolic, colour, antioxidant and aroma of Cabernet Sauvignon red wine from Xinjiang (China)

Huan Wang, Yuanyuan Miao, Xiaoyu Xu, PipingYe, Huimin Wu, Bin Wang *, and Xuewei Shi *

Food college, Shihezi University, Shihezi 832000, Xinjiang Uygur Autonomous Region, P. R. China

* Corresponding Author:

Xuewei Shi, Email: shixuewei@shzu.edu.cn

Bin Wang, Email: binwang0228@shzu.edu.cn

Tel: 86-0993-2058093.

Supporting information

Figure. S1. The key volatile compounds in different wine samples (OAV>1). 2

Table. S1. Physicochemical indexes of different grape varieties. 2

Table. S2. Volatile compounds identified in this work and their aroma parameters. 3

Table. S3. Concentration (μg/L, mean ± SD) of the volatile compounds in different blending treatment wines. 5

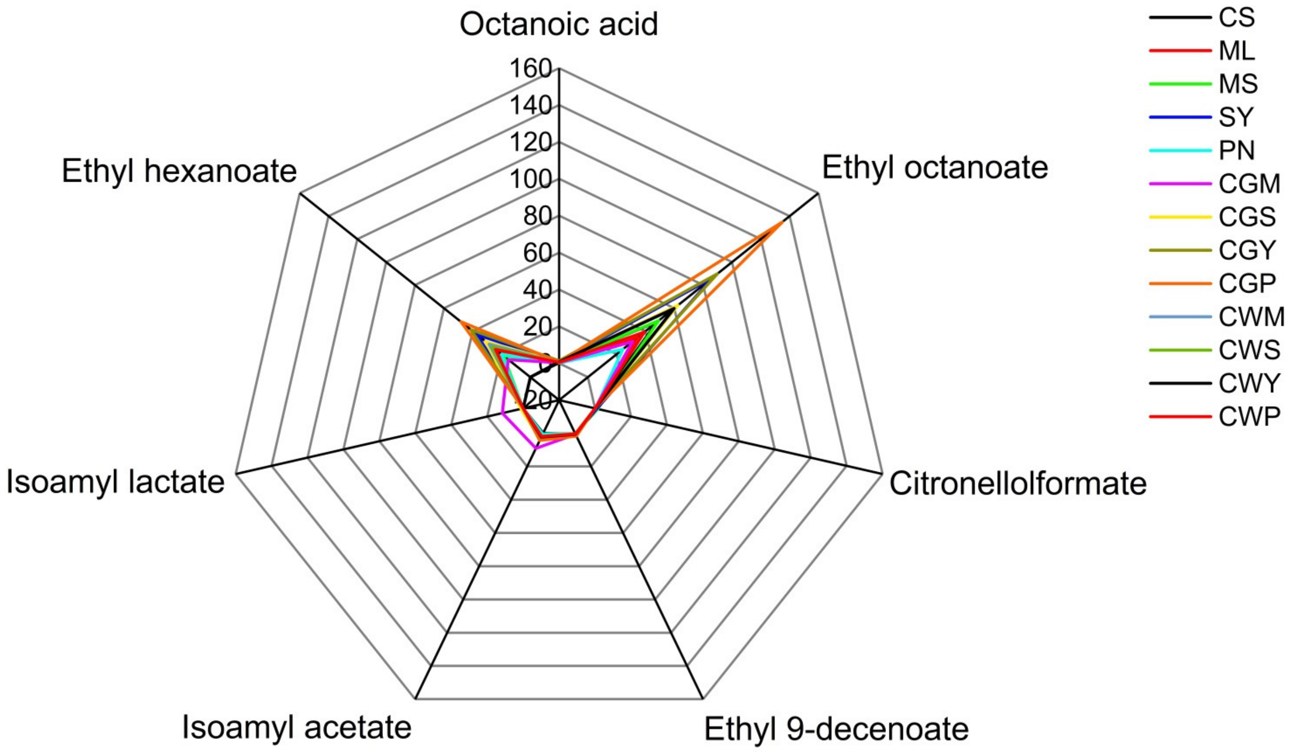

Figure S1. The key volatile compounds in different wine samples (OAV>1)

Table S1 Basic physicochemical parameters of different grape varieties

**Sample**	**Soluble solids（°Brix）**	**Total sugar（g/L）**	**Total acid（g/L）**	**pH**
CS	24.6±2.6a	168.33±28.39a	6.75±0.91a	4.31±0.81a
ML	23.2±3.6ab	181.36±15.36b	6.61±0.69a	4.26±0.21a
MS	21.7±2.1ab	172.39±11.04a	6.55±1.28a	4.13±0.31a
SY	20.5±0.9b	166.21±33.49a	6.41±1.12a	4.09±0.28a
PN	24.5±1.1ab	175.78±30.65a	6.77±0.61a	4.42±0.78a

[a]Values are expressed as mean ± standard deviation (n = 3). Different letters in a column denote significant differences among samples ($P < 0.05$, Tukey's HSD test).

Table S2 Volatile compounds identified in this work and their aroma parameters

Compounds	RI	Odor threshold (μg/L)	Aroma series	OAV	Odor description
**Ester**					
Ethyl Acetate	890	7500[6]	1[12]	<0.1	Fruity, nail polish, balsamic
Ethyl butyrate	1028	20[4]	1[11]	0.1-1	Strawberry, apple, banana
Isoamyl acetate	1123	30[10]	1[6]	>1	Banana
Ethyl hexanoate	1232	5[3]	1[4]	>1	Banana,green apple
Leaf salicylate	1315	11000[11]	2[12]	>1	Floral
3-Hexenyl isobutyrate	1385				
Ethyl octanoate	1429	5[1]	1,2[2]	>1	Fruity, banana,pear
Ethyl nonanoate	1535	1300[7]	1,8[2]	<0.1	Waxy, fruity
Isoamyl lactate	1572	5[7]	5[1]	>1	Caramel
Methyl caprinate	1591	3[9]		0.1-1	
Citronellolformate	1617	5[2]	2[11]	0.1-1	Flowery
Ethyl decanoate	1639	200[4]	1,8[7]	0.1-1	Fruity, fatty
3-Methylbutyl octanoate	1659	125[12]	1[10]	<0.1	Pineapple, coconut
Ethyl 3-hydroxybhexanoate	1675				
Ethyl benzoate	1678				
Diethyl succinate	1682	500000[11]	1[5]	>1	Fruity, melon
Ethyl 9-decenoate	1703	100[5]	1[4]	0.1-1	Fruity
Dibutyl adipate	1730				
n-Propyl benzoate	1745	6550[1]		>1	
Ethyl 2-phenylacetate	1798	250[2]		<0.1	
Phenethyl acetate	1830	250[10]	2[7]	<0.1	Floral
Ethyl laurate	1848	1500[5]	1,2,9[8]	<0.1	Sweet, floral, fruity, cream
Dibutyl phthalate	2630				
**Alcohol**					
Ethyl alcohol	930	5[8]		>1	
Isoamylol	1197	30000[5]	7,5,8[9]	<0.1	Whiskey, nail polish
1-Hexanol	1339	8000[6]	3[11]	<0.1	Herbaceous, grass, woody
1-Heptanol	1461	200[5]	1,6[1]	<0.1	Lemon, orange, copper
1-Octanol	1567	120[10]	1,2[8]	<0.1	Citrus, rose

1-Nonanol	1661	58[4]	3[2]	0.1-1	Green
3-Methylthiopropanol	1726	500[2]	7,9[3]	<0.1	Boiled potato, rubber
6-Nonen-1-ol	1727				
1-Decanol	1757	400[1]	2,8[7]	<0.1	Orange, flowery, fatty
Citronellol	1770	100[9]	2[6]	<0.1	Rose
Benzyl alcohol	1892	200000[7]	4[11]	>1	Almond
Phenylethyl alcohol	1928	10000[8]	2[8]	0.1-1	Floral, rose
**Acid**					
4-Hydroxybutyric acid	933				
Acetic acid	1463	200000[5]	7[5]	<0.1	Acid, fatty
Isobutyric acid	1590	200000[9]	8[3]	>1	Fatty
2-Methylhexanoic acid	1757				
Hexanoic acid	1857	420[2]	3,7[5]	<0.1	Leafy, wood, varnish
Octanoic acid	2075	500[4]	4,8[7]	0.1-1	Butter, almond
Nonanoic acid	2171				
Decanoic acid	2279	1000[1]	5[9]	<0.1	Caramel
**Aldehyde**					
Decanal	1495	1000[1]	3[11]	<0.1	Grassy
Benzaldehyde	1534	800[10]	4[8]	<0.1	Almond
2,5-Dimethylbenzaldehyde	1705				
Benzaldehyde, 2,4-dimethyl	1742				
**Phenol**					
2,4-Di-tertbutylphenol	2280	200[4]	7[10]	<0.1	Alkylphenol odor

[a]Aroma series 1 = fruity, 2 = floral, 3 = herbaceous (or vegetal), 4 = nutty, 5 = caramel, 6 = earthy, 7 = chemical, 8 = fatty, 9 = roasted.

[b]OAV was calculated by dividing concentration by the odor threshold value of the compound. The scope of OAV is shown but not the speci fic value. The nf represented not found.

[c] [1] (Anon et al., 2014)), [2] (Daniel et al., 2019), [3] (Garcia et al., 2011), [4] (Jian C et al., 2007), [5] (Lorenzo et al., 2008), [6] (López et al., 2003), [7] (Huang et al., 2021), [8] (Peng et al., 2005), [9] (Petropulos et al., 2014), [10] (Siebert et al., 2018)., [11] (Sánchez et al., 2017), [12] (Yilmaztekin et al., 2015).

Table S3 Concentration (μg/l, mean ± SD) of the volatile compounds in different blending treatment wines

	Single wine					Co-grapes				Co-wines			
Compound	CS	ML	MS	SY	PN	CGM	CGS	CGY	CGP	CWM	CWS	CWY	CWP
**Ester**													
Ethyl Acetate	nd	nd	nd	63.5±8.04e	196.57±13.26c	281.35±27.85a	215.17±11.56b	29.71±2.18f	157.63±13.73d	75.89±5.77e	22.84±3.11f	nd	68.92±3.94g
Ethyl butyrate	nd	16.41±1.23a	nd	2.9±0.37c	nd	nd	5.52±0.86b	nd	nd	0.6±0.08d	5.65±0.66b	nd	nd
Isoamyl acetate	nd	34.94±4.79e	115.87±12.32b	111.68±15.01b	13.96±1.41f	271.04±32.14a	108.44±7.22b	83.62±5.23c	125.86±18.82b	54.23±3.56de	52.74±3.33de	64.87±1.35cd	55.30±5.56d
Ethyl hexanoate	nd	120±15.93c	92.81±13.03de	185.73±32.29b	99.59±2.26c	76.33±10.57e	155.39±10.41b	205.54±10.35b	240.55±20.86a	142.87±21.81c	131.72±4.96c	nd	120.85±13.01cd
Leaf salicylate	0.61±0.03a	nd	nd	nd	nd	nd	nd	nd	nd	nd	nd	nd	nd
3-Hexenyl isobutyrate	nd	1.22±0.10a	nd	nd	nd	nd	nd	nd	nd	nd	nd	nd	nd
Ethyl octanoate	241.36±23.01cde	172.72±20.74efg	245.42±19.48de	431.89±53.26b	121.15±14.08g	155.31±16.25fg	312.43±14.99cd	448.89±74.54b	674.47±102.25a	195.22±30.18ef	195.45±20.09def	298.98±29.53c	195.97±16.71def
Ethyl nonanoate	0.79±0.07b	1.56±0.20a	0.19±0.03c	nd	nd	nd	nd	nd	nd	nd	nd	1.55±0.12a	nd
Isoamyl lactate	nd	nd	nd	nd	nd	57.91±2.38a	6.26±0.74b	nd	nd	nd	nd	nd	nd
Methyl caprinate	nd	nd	nd	nd	nd	nd	nd	nd	nd	nd	1.28±0.22b	2.91±0.42a	0.61±0.03c
Citronellolformate	nd	nd	7.53±0.83a	4.36±0.2b	0.93±0.14c	nd	nd	nd	nd	nd	nd	nd	nd
Ethyl decanoate	nd	nd	nd	nd	nd	nd	nd	nd	nd	nd	106.21±12.94c	190.34±14.83a	144.31±9.64b

3-Methylbutyl octanoate	6.02±0.45b	2.44±0.31d	2.55±0.41d	2.25±0.05d	nd	3.63±0.71c	4.35±0.74c	6.49±0.91b	7.93±0.85a	1.08±0.06f	1.93±0.02de	4.44±0.31c	1.64±0.19ef
Ethyl 3-hydroxybhexanoate	0.98±0.21c	nd	1.08±0.18b	nd	nd	nd	nd	nd	nd	1.07±0.08bc	2.53±0.09a	nd	nd
Ethyl benzoate	4.17±0.09b	2.79±0.48d	1.62±0.19e	2.05±0.3e	2.46±0.16d	3.93±0.63c	nd	nd	5.33±0.33a	2.93±0.16d	1.98±0.39e	2.88±0.17d	2.98±0.08d
Diethyl succinate	3.14±0.14cd	0.71±0.13h	2.21±0.36g	4.11±0.17a	nd	1.35±0.20g	2.88±0.17ef	3.14±0.26def	3.94±0.67bc	3.66±0.46bc	3.43±0.19de	4.4±0.33ab	2.95±0.29de
Ethyl 9-decenoate	66.01±4.08d	70.66±11.51c	27.43±0.87ef	53.99±9.12d	26.37±1.65f	52.77±8.01d	31.49±3.21ef	105.64±6.66b	161±12.45a	32.62±3.41ef	27.39±3.56ef	51.99±0.36d	36.09±6.61e
Dibutyl adipate	nd	nd	0.58±0.07c	nd	nd	nd	nd	3.41±0.12b	nd	nd	nd	8.19±1.35a	nd
n-Propyl benzoate	nd	nd	nd	nd	0.39±0.03a	nd	nd	nd	nd	nd	nd	nd	nd
Ethyl 2-phenylacetate	4.44±0.5ab	nd	3.34±0.06c	3.41±0.13c	nd	3.78±0.73bc	nd	nd	nd	4.04±0.47ab	3.19±0.34c	4.75±0.92a	4.31±0.49ab
Phenethyl acetate	111.62±12.42c	20.89±1.35g	82.17±1.82cde	45.54±4.43f	14.62±2.47g	150.84±25.07a	56.85±3.54f	121.14±11.19b	91.65±11.14cde	82.14±6.51de	75.2±7.56e	93.3±14.69cde	106.42±2.36cd
Ethyl laurate	33.25±2.40c	93.03±9.82a	31.64±3.01c	9.23±1.27gh	11±1.03fgh	39.75±7.24b	7.20±0.57gh	19.45±0.92de	21.43±1.18d	nd	5.86±0.12hi	16.81±3.03ef	13.79±1.92efg
Dibutyl phthalate	5.94±0.65b	nd	5.28±0.82c	nd	nd	3.29±0.57d	nd	nd	nd	nd	3.66±0.31d	7.47±0.95a	7.11±0.12ab
**Alcohol**													
Ethyl alcohol	2136.88±705.64c	2674.96±80.45b	557.39±80.62gh	432.92±27.86h	1334.38±53.13d	2822.64±217.4a	1060.03±216.11ef	803.48±16.50gh	1702.84±67.78c	1346.58±41.25d	651.29±64.31gh	965.6±93.45fg	1288.81±76.58de
Isoamylol	1565.27±109.53bc	1605.62±0.59cd	1387.12±176.27d	557.95±10.97i	782.43±87.45gh	3596.05±149.77a	1662.62±107.85bc	1270.81±143.81de	2222.2±152.58b	887.76±47.35fg	797.99±86.19h	nd	1209.43±172.39ef
1-Hexanol	51.05±7.3	11.08±1.	12.65±1.	185.73±	31.8±5.	52.26±5.2	19.73±3.	18.86±0.	40.66±2	31.82±0.6	29.42±1.	34.25±2.	43.75±5.

	9b	99f	62f	31.44a	36cde	5b	05ef	88ef	.67bc	7cdef	82cdef	88bcd	21bc
1-Heptanol	nd	nd	3.83±0.49d	4.33±0.33cd	4.09±0.52d	nd	9.41±0.13b	9.67±1.43b	12.12±1.22a	4.85±0.55cd	3.67±0.35cd	5.29±0.74c	5.01±0.34c
1-Octanol	11.62±1.73ab	nd	7.43±1.11c	12.51±2.14a	6.41±0.45de	4.5±0.45e	6.83±1.13cd	nd	10.48±0.78ab	nd	6.7±0.59de	nd	10.43±1.52b
1-Nonanol	14.95±1.64bc	7.8±0.28ef	7.63±1.4f	11.5±1.01cd	7.57±0.46f	9±1.3def	8.38±0.49def	nd	11.62±0.97de	15.11±2.47b	13.13±1.33bc	16.25±2.51ab	18.33±1.89a
3-Methylthiopropanol	3.03±0.08d	nd	2.36±0.22e	2.03±0.25e	nd	5.95±0.69b	3.55±0.04d	5.97±0.34a	4.02±0.66c	3.25±0.14d	3.46±0.46d	4.21±0.31c	2.01±0.18e
6-Nonen-1-ol	0.82±0.07b	nd	nd	0.31±0.02c	nd	nd	nd	2.44±0.31a	nd	nd	1.13±0.13b	2.57±0.41a	nd
1-Decanol	5.84±0.68c	nd	nd	nd	nd	nd	nd	nd	nd	nd	3.72±0.23d	7.05±1.01b	7.15±0.89a
Citronellol	nd	nd	nd	nd	nd	nd	nd	6.89±0.83a	nd	nd	nd	6.73±0.45a	nd
Benzyl alcohol	nd	nd	nd	5.17±0.51f	21.59±1.08a	nd	nd	nd	15.43±2.32c	12.46±1.35d	9.41±1.62e	11.81±1.89d	20.46±1.14b
Phenylethyl alcohol	1483.55±170.06c	447.3±50.79f	1452.39±147.98cd	735.35±154.44e	434.64±44.19f	2019.97±305.7b	1725.4±134.11cd	1481.05±26.87cd	1585.6±96.35cd	1645.88±127.53cd	1265.92±55.11d	1478.67±196.58c	1552.5±39.16cd
**Acid**													
4-Hydroxybutyric acid	2.31±0.49ef	3.11±0.3d	1.91±0.03fg	1.21±0.07g	6.15±0.49a	6.05±0.7a	4.47±0.3b	2.55±0.25de	3.82±0.43c	4.33±0.3bc	2.37±0.07de	3.11±0.28d	2.83±0.42d
Acetic acid	33.18±3.38de	72.63±7.1b	27.22±2.21fg	14.16±1.76h	55.25±0.84c	114.31±2.27a	22.84±3.42g	36.35±5.69d	19.27±2.29g	32.70±1.7d	23.84±2.03g	20.12±2.89g	29.59±1.32ef
Isobutyric acid	9.3±1.08b	nd	3.55±0.44d	2.39±0.09e	nd	9.39±0.53b	3.52±0.44de	5.59±1.09c	nd	nd	5.40±0.62c	5.97±0.44c	3.58±0.53d
2-Methylhexanoic acid	13.64±1.5a	nd	nd	nd	nd	nd	nd	11.84±1.95ab	nd	6.99±1.22d	10.10±0.95b	9.29±0.57c	8.86±1.16c

Hexanoic acid	38.07±0.65ef	6.98±0.46g	30.81±3.33f	43.41±6.17cd	6.15±0.48g	34.6±5.42ef	51.31±6.48c	81.75±9.61a	39.07±4.36de	nd	40.85±3.96de	53.93±6.47b	38.12±3.42de
Octanoic acid	230.11±10.53ef	21.22±4.2h	236.29±36.07de	324.56±19.38c	26.1±2.79h	117.82±20.29g	235.86±26.27d	676.11±15.35a	310.81±31.78c	176.57±27.18f	212.51±34.33def	383.92±42.54b	255.67±14.57de
Nonanoic acid	nd	nd	1.26±0.2b	nd	nd	nd	nd	5.22±0.62a	nd	nd	0.81±0.08c	nd	nd
Decanoic acid	66.65±12.94c	4.46±0.29f	51.69±0.54c	23.99±3.97de	nd	1.49±0.11f	9.04±1ef	113.41±20.83a	nd	23.34±3.17de	38.03±4.46d	79.98±14.41b	56.51±10.91c
**Aldehyde**													
Decanal	nd	nd	nd	3.5±0.36b	nd	nd	nd	3.82±0.43b	nd	nd	6.21±0.87a	5.41±0.83a	3.88±0.33b
Benzaldehyde	3.92±0.42cd	2.49±0.08e	2.69±0.31e	nd	3.06±0.46de	10.91±1.16a	4.3±0.75c	nd	5.43±0.35b	nd	nd	nd	nd
2,5-Dimethylbenzaldehyde	81.2±5.53c	15.56±1.15e	nd	56.39±11.36d	nd	nd	79.42±7.11c	95.23±10.21b	104.5±15.54a	62.14±11.93cd	nd	nd	69.25±5.19c
Benzaldehyde, 2,4-dimethyl	nd	nd	72.63±12.12a	nd	26.19±2.65c	53.46±5.74b	nd	nd	nd	nd	49.31±4.68b	61.29±7.94b	nd
**Phenol**													
2,4-Di-tertbutylphenol	35.83±6.44b	nd	22.16±1.94cd	13.96±1.35e	nd	25.39±1.46c	11.83±0.92e	2.47±0.22f	nd	19.15±3.59cd	17.85±1.53d	36.52±1.95a	32.17±2.47b
**Other**													
1-Phenyl-1,2-propanediol	9.36±1.56a	4.23±0.35c	5.91±0.91b	nd	nd	nd	nd	nd	nd	nd	nd	nd	nd
1,3-Pentanediol,2,2,4-trimethyl-,diis	3.4±0.08d	nd	nd	nd	nd	nd	nd	8.94±0.95a	5.38±0.29b	nd	nd	4.77±0.24c	3.85±0.31c
Homosalate	nd	nd	0.52±0.06g	5.74±0.24g	2.19±0.01ef	nd	nd	3.19±0.10e	8.46±1.17c	1.84±0.13ef	3.93±0.22de	25.93±3.11a	14.63±0.91b
Allyl phenylacetate	0.4±0.03a	nd	nd	nd	nd	nd	nd	nd	nd	nd	nd	0.29±0.0	nd

												3b	
Erythro-1-Phenyl-1, 2-dihydroxypropan	nd	nd	5.91±0.2 9b	nd	nd	7.53±0.61 a	5.89±0.3 7c	6.71±0.9 7ab	nd	nd	nd	nd	nd

[a]Data are expressed as the means ± standard (n = 3). The different lowercase letters in each row indicate a signi ficant difference between the samples (P < 0.05). nd, not detected.
